# A case of granulomatosis with polyangiitis mimicking lung malignancy

**DOI:** 10.1002/rcr2.824

**Published:** 2021-08-09

**Authors:** Michael Han, Corinna Pan

**Affiliations:** ^1^ Department of Respiratory and Sleep Medicine Sutherland Hospital Sydney New South Wales Australia

**Keywords:** granulomatosis with polyangiitis, lung nodules

## Abstract

Patients with granulomatosis with polyangiitis (GPA) may present with varied manifestations including pulmonary masses and nodules. We report the case of a 45‐year‐old man presenting with cough, haemoptysis and weight loss in the context of a 20 pack‐year past smoking history. Computed tomography (CT) of the chest and positron emission tomography/CT scan demonstrated two right upper lobe masses, bilateral lung nodules and mediastinal lymphadenopathy, with increased fluorodeoxyglucose avidity. Endobronchial and CT‐guided lung biopsy demonstrated granulomatous inflammation and elevated c‐ANCA/PR3 confirmed the diagnosis of GPA. The patient received induction therapy with methylprednisolone and rituximab with good clinical response. Our case highlights the importance of considering a wide range of differentials in patients with lung masses/nodules, including autoimmune pathologies.

## Graphical abstract

Granulomatosis with polyangiitis (GPA) may present with varied manifestations including lung nodules/masses mimicking malignancy. We present the case of a patient presenting with cough, haemoptysis and constitutional symptoms on a background of significant past smoking history, with multiple lung nodules and mediastinal lymphadenopathy found on chest computed tomography. Biopsy showed granulomatous inflammation and elevated c‐ANCA/PR3 confirmed the diagnosis of GPA.

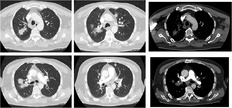

## INTRODUCTION

Granulomatosis with polyangiitis (GPA) represents one of a number of pulmonary vasculidities which can present with varied clinical, radiographic and laboratory manifestations. The most common radiographic presentation of GPA is with pulmonary masses and nodules, which are often multiple and cavitating.^1^ This presentation may mimic lung malignancy, including amongst patients with diagnosed GPA, which has been shown to increase malignancy risk including for lung cancer.^2^

We report a case of GPA mimicking lung cancer in a 45‐year‐old man presenting with cough, haemoptysis and weight loss on a background of a significant past smoking history.

## CASE REPORT

A 45‐year‐old male presented with 4 weeks of exertional dyspnoea and cough, with 2 weeks of intermittent haemoptysis. He reported associated anorexia and weight loss over this period. Outpatient treatment including three courses of oral antibiotic therapy did not improve his symptoms, prompting presentation to our hospital. His medical background included a 20 pack‐year past smoking history, hypertension, gout, vitamin B12 deficiency and intellectual disability. On presentation, his only regular medication was irbesartan 150 mg daily. He lived at home and reported no known tuberculosis or occupational exposures.

On examination, he appeared well with no evidence of respiratory distress. His vital signs were: blood pressure 127/78 mmHg, heart rate 98 beats per minute, respiratory rate 20 breaths per minute, oxygen saturation 99% (on room air) and temperature 37.8°C. Chest auscultation revealed equal air entry bilaterally with no adventitious sounds. Cardiovascular, abdominal and lower limb examinations were unremarkable.

**FIGURE 1:**
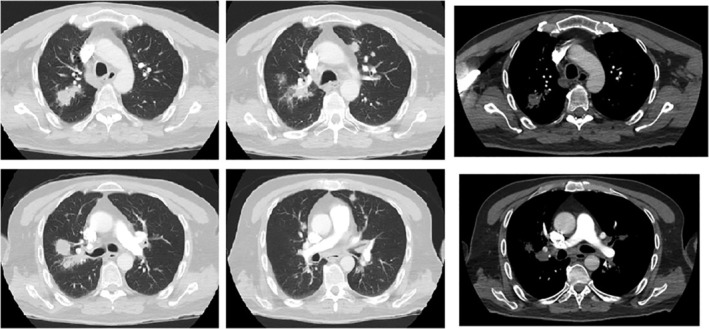
Computed tomography pulmonary angiogram showing two right upper lobe spiculated lung masses with multiple bilateral pulmonary nodules and mediastinal lymphadenopathy

His pathology results at presentation are shown in Table 1. A computed tomography (CT) pulmonary angiogram was performed, which showed two right upper lobe (RUL) spiculated masses, as well as multiple bilateral pulmonary nodules and mediastinal lymphadenopathy (Figure 1).

**TABLE 1:** Laboratory findings at admission, following initial induction therapy with methylprednisolone and rituximab (completed on Day 55 of admission), and prior to discharge (on Day 117 of admission)

Investigations	At admission (Day 0)	Following induction therapy (Day 55)	Before discharge (Day 117)
Haemoglobin (g/L)	107	102	138
White cell count (×10^9^/L)	10.7	9.0	7.2
Platelet count (×10^9^/L)	492	337	214
C‐reactive protein (mg/L)	114	6	3
Erythrocyte sedimentation rate (mm/h)	99		
ANCA	c‐ANCA, titre 1:160	c‐ANCA, titre 1:10	
PR3 (U/mL)	45.0	1.8	
MPO (U/mL)	0.2	<0.1	
Creatinine (μmol/L)	75	71	77
Urine microscopy	Hyaline and granular casts No dysmorphic RBC No haematuria or proteinuria		
Protein (g/L)	77	54	54
Albumin (g/L)	25	21	30

Abbreviations: ANCA, antineutrophil cytoplasmic antibody; MPO, myeloperoxidase antineutrophil cytoplasmic antibody; PR3, proteinase 3; RBC, red blood cell.

**FIGURE 2:**
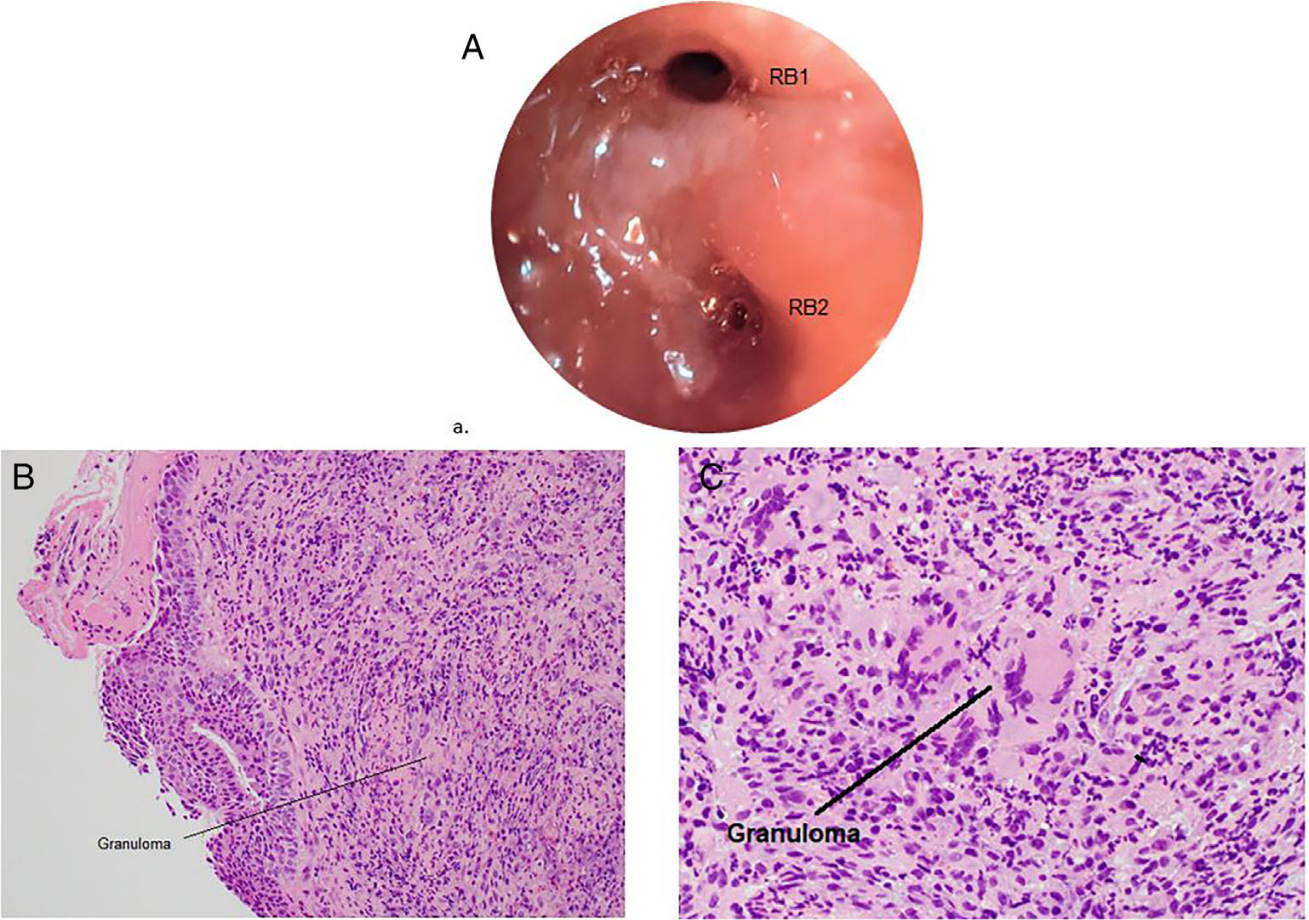
(A) Endobronchial view showing partial obstruction of apical (RB1) and posterior (RB2) segmental bronchi of the right upper lobe. (B) Endobronchial biopsy histopathology specimen (haematoxylin and eosin staining, low magnification) demonstrating bronchial mucosa with inflamed granulation tissue. (C) Endobronchial biopsy histopathology specimen (haematoxylin and eosin staining, high magnification) demonstrating granuloma

The patient was admitted for management of haemoptysis and investigation of presumed metastatic malignancy. He was commenced on intravenous (IV) ceftriaxone and azithromycin to treat for potential community‐acquired pneumonia and administered nebulized tranexamic acid for haemoptysis. On Day 2 of admission, flexible bronchoscopy was performed, which showed a soft tissue mass causing partial obstruction of the RUL apical and posterior segmental bronchi with active haemorrhagic oozing from this site (Figure 2). Adrenaline (3 ml, 1:100,000) was instilled for haemostasis. Endobronchial biopsies, brushings and washings were obtained from this site. Positron emission tomography (PET)/CT scan was performed which showed diffusely increased laryngeal uptake [standardized uptake value (SUV) 13.5] in the context of recent bronchoscopy, two RUL spiculated masses (SUV 15.5 in larger mass), scattered bilateral fluorodeoxyglucose (FDG)‐avid lung nodules and FDG‐avid mediastinal lymphadenopathy (SUV 4.4) with no other FDG‐avid lesions. CT of the brain showed extensive inflammatory paranasal sinus disease with no intra‐cranial lesions. Given a lack of symptoms of sinus disease, this radiological finding was not further investigated at that stage. While awaiting results from the bronchoscopic biopsies, stereotactic radiotherapy (single fraction 6 Gy) was administered to the RUL mass for the management of ongoing haemoptysis, with good effect.

On Day 5 of admission, results from bronchoscopic histopathology showed acutely inflamed granulation tissue with poorly formed granulomas (Figure 2). Bacterial, fungal and mycobacterial microbiological testing revealed no abnormalities.

Due to concerns about potentially non‐representative sampling from the bronchoscopy specimen, a CT‐guided lung biopsy was performed, which showed chronic inflammation with fibrosis and occasional poorly formed granulomas. Vasculitis serology was performed which showed positive cytoplasmic antineutrophil cytoplasmic antibodies (c‐ANCA) with elevated proteinase 3 (PR3) antibodies (Table 1). A subsequent diagnosis of GPA was made and the patient received induction therapy with IV pulse methylprednisolone (3 days of 500 mg/day) and rituximab (two doses of 1 g) therapy. Treatment was complicated by the development of invasive aspergillosis requiring management with 6 weeks of posaconazole, as well as one episode of disease relapse with rising inflammatory markers and increased cough requiring a further 5‐day course of IV methylprednisolone. The patient improved clinically with resolution of cough and normalization of inflammatory markers and ANCA titres (Table 1). He is now receiving a weaning course of prednisone and ongoing rehabilitation prior to discharge.

## DISCUSSION

We present a case of GPA mimicking lung cancer in a patient with significant smoking history who presented with cough, haemoptysis and weight loss. GPA is a small vessel vasculitis which classically presents with a triad of pulmonary, upper airways and renal manifestations. Pulmonary involvement occurs in 90% of patients and varies in severity from asymptomatic disease to diffuse alveolar haemorrhage.^3^ The most common radiographic presentation of GPA is of pulmonary masses and nodules which are often multiple and cavitating, although a range of other pulmonary and pleural manifestations may occur.^1^ PET/CT scanning in GPA may be useful in identifying pulmonary involvement and guiding decisions regarding biopsy site but cannot distinguish between inflammatory and malignant lesions. Reduction in FDG uptake post‐treatment has been reported, but the role of PET/CT scans in monitoring treatment response requires further delineation.^4^ Diagnosis is made based on a combination of clinical, radiological and immunological findings, with biopsy results supporting the diagnosis.^3^, ^5^ The radiographic lesions of GPA may mimic malignancy, with spiculation and invasion of the surrounding structures reported.^6^ Furthermore, GPA is itself associated with an increased risk of malignancy, including the risk of pulmonary malignancy.^2^ Conversely, coexisting granulomatous inflammation may be seen on histopathology specimens amongst patients with lung cancer, possibly reflecting previous mycobacterial infection or granulomatous reaction to malignancy.^7^ In our case, our clinical suspicion of malignancy remained sufficiently high following the initial lung biopsy that a second biopsy was pursued, which assisted in confirming the diagnosis of GPA.

In our patient, radiotherapy was administered prior to the diagnosis of GPA for management of haemoptysis, with good effect. While successful use of radiation therapy to treat haemoptysis from aspergilloma complicating perinuclear antineutrophil cytoplasmic antibody (p‐ANCA) positive vasculitis has been reported,^8^ there have been no previously reported cases of radiotherapy use to control haemoptysis directly caused by pulmonary vasculitis. Currently, the short‐term haemostatic effectiveness of radiotherapy is thought to be due to increased platelet adhesion to vascular endothelium, while long‐term effect could be explained by vessel fibrosis.^9^ In cancer patients, radiotherapy also causes tumour shrinkage. Given its proposed mechanisms, it is perhaps not surprising that we were able to achieve acute haemostasis with our case. The effects of radiotherapy in achieving haemostasis can occur within 24–48 h of therapy^10^ and in our case, occurred before the initiation of immunosuppression therapy, further supporting this hypothesis. Nonetheless, the medium‐ and long‐term effect of radiotherapy in maintaining haemostasis is difficult to predict, and this interesting observation will require more data to assess its validity and utility.

Overall, our case highlights the need to consider a broad range of differentials, including non‐malignant pathologies such as vasculitis, in the investigation of patients presenting with pulmonary masses/nodules. Our case suggests a possible role for the use of radiation therapy to acutely control haemoptysis from vasculitis but more data are required.

## CONFLICT OF INTEREST

None declared.

## ETHICS STATEMENT

Appropriate written informed consent was obtained for publication of this case report and accompanying images.

## AUTHOR CONTRIBUTIONS

Dr Michael Han obtained clinical data, performed literature review and wrote this manuscript. Dr Corinna Pan provided critical input into manuscript preparation. Both authors reviewed and approved the final manuscript.
